# QTL Mapping for Ovary- and Fruit-Related Traits in *Cucumis sativus-C. hystrix* Introgression Line IL52

**DOI:** 10.3390/genes14061133

**Published:** 2023-05-23

**Authors:** Yuhui Wang, Yu Fang, Shixiong Ning, Lei Xia, Jinyi Zhan, Zhilong Yang, Chunyan Cheng, Qunfeng Lou, Ji Li, Jinfeng Chen

**Affiliations:** State Key Laboratory of Crop Genetics & Germplasm Enhancement and Utilization, College of Horticulture, Nanjing Agriculture University, Nanjing 210095, China; yuhui_wang@njau.edu.cn (Y.W.); 2020104048@stu.njau.edu.cn (Y.F.); 2021104049@stu.njau.edu.cn (S.N.); 2019204026@njau.edu.cn (L.X.); 2022104070@stu.njau.edu.cn (J.Z.); 2020804135@stu.njau.edu.cn (Z.Y.); chunyancheng@njau.edu.cn (C.C.); qflou@njau.edu.cn (Q.L.); liji1981@njau.edu.cn (J.L.)

**Keywords:** cucumber, QTL mapping, ovary hypanthium neck, ovary size, fruit size, introgression line

## Abstract

IL52 is a valuable introgression line obtained from interspecific hybridization between cultivated cucumber (*Cucumis sativus* L., 2n = 14) and the wild relative species *C. hystrix* Chakr. (2n = 24). IL52 exhibits high resistance to a number of diseases, including downy mildew, powdery mildew, and angular leaf spot. However, the ovary- and fruit-related traits of IL52 have not been thoroughly investigated. Here, we conducted quantitative trait loci (QTL) mapping for 11 traits related to ovary size, fruit size, and flowering time using a previously developed 155 F_7:8_ RIL population derived from a cross between CCMC and IL52. In total, 27 QTL associated with the 11 traits were detected, distributed on seven chromosomes. These QTL explained 3.61% to 43.98% of the phenotypic variance. Notably, we identified a major-effect QTL (*qOHN4.1*) on chromosome 4 associated with the ovary hypanthium neck width and further delimited it into a 114-kb candidate region harboring 13 candidate genes. Furthermore, the QTL *qOHN4.1* is co-localized with the QTL detected for ovary length, mature fruit length, and fruit neck length, all residing within the consensus QTL *FS4.1*, suggesting a plausible pleiotropic effect.

## 1. Introduction

Cucumber (*Cucumis sativus* L.) is a globally significant commercial vegetable that provides essential nutrients and contributes to improved dietary health. Cucumbers can be consumed in various forms, including immature or mature and fresh or processed [1]. The cucumber fruit’s shape and size are two crucial breeding traits that are adapted to meet different market preferences, packaging and shipment requirements, and processing specifications. For example, in northern China, fresh market cucumbers are often spiny fruits with a length of 25–30 cm, while in the US, pickling cucumbers have a length-by-diameter (L/D) ratio of approximately 3.0, ideal for fitting into glass jars [2]. The genetic inheritance and corresponding quantitative trait loci (QTL) of cucumber fruit size have been extensively investigated in populations derived from crosses between different market-class cucumbers (e.g., [3,4,5,6]). A total of 185 QTL for fruit-related traits have been identified, which could be further summarized and established as 14 consensus Fruit Size (FS) QTL (*FS1.1*, *FS1.2*, *FS2.1*, *FS2.2*, *FS3.1*, *FS3.2*, *FS3.3*, *FS4.1*, *FS5.1*, *FS5.2*, *FS6.1*, *FS6.2*, *FS6.3*, and *FS7.1*) [1]. Some of the underlying causal genes for these QTL were identified, such as *CsTRM5* for *FS2.1* [7,8] and *CsCRC* for *FS5.2* [9]. However, the majority of these studies mainly focus on the immature fruit (commercial harvest stage) or mature fruit, with limited investigation at the ovary stage. Although there is a certain degree of correlation in length and diameter between the ovary and fruit, distinct genetic mechanisms are involved in regulating cell division and expansion during the transition from ovary to mature fruit, characterized by spatial-temporal gene expression patterns [2,10]. Moreover, certain ovary traits, such as the hypanthium, have not yet been fully investigated.

The female flower of cucumber is an epigynous flower with an inferior ovary. The entire ovary is embedded in the sunken receptacle, while the hypanthium is fused to the gynoecium. The free sepals, petals, and stigma appear to be attached to the superior part of the gynoecium. As the ovary grows, the hypanthium eventually develops into the exocarp of the cucumber fruit. The entire ovarian-stage hypanthium is hourglass-shaped, with a slender neck connecting the superior corolla and inferior ovary ([11]; Figure S1). Therefore, we termed the narrow neck the ovary hypanthium neck (OHN). In cucumber breeding practices, a corolla with a broader OHN makes hand pollination simpler, but a corolla with a narrow OHN is delicate. Additionally, as the ovary and fruit mature, the corolla of spontaneous parthenocarpy fruits or pollinated fruits often dries up and falls off, leaving a fruit scar at the bottom that diminishes the fruit’s aesthetic appeal. However, the genetic inheritance of OHN has not yet been studied.

IL52 is an introgression line derived from interspecific hybridization between cultivated cucumber and the wild relative species *Cucumis hystrix* (2n = 24) [12]. It exhibits high resistance to downy mildew, powdery mildew, and angular leaf spot (ALS), traits that have been extensively studied for their genetic inheritance [13,14,15]. Notably, IL52’s ovaries possess a relatively thin OHN and exhibit distinct horticultural traits, such as a round fruit apex, compared with the pointed fruit apex of cucumbers from the northern China market class [16]. Consequently, a thorough investigation into the genetic inheritance of ovary and fruit appearance quality traits in IL52 would broaden its applicability in cucumber breeding.

In this study, we conducted QTL mapping for ovary- and fruit-related traits using a previously developed F_7:8_ RIL population comprising 155 individuals derived from the cross between CCMC and IL52. The QTL mapping analysis revealed a total of 27 QTL for 11 traits. Furthermore, we delimited the candidate region for *qOHN4.1* and proposed the best candidate genes which might have pleiotropic effects for the consensus *FS* QTL *FS4.1*.

## 2. Materials and Methods

### 2.1. Plant Materials

The cultivated cucumber CMCC and introgression line IL52 were used to develop segregating recombinant inbred lines (RILs) populations containing 155 F_7:8_ individuals. The CCMC is a typical northern China fresh market-type cucumber with a wider ovary hypanthium neck (OHN) and slim, spiny, and dark green immature fruits that are susceptible to a number of diseases, such as downy mildew (DM), powdery mildew (PM), and angular leaf spot (ALS). IL52 is derived from interspecific hybridization between cucumber and the wild relative species *Cucumis hystrix* [13,14]. IL52 bears narrow OHN, short, relatively smooth, and light green immature fruits resistant to DM, PM, and ALS diseases.

### 2.2. Phenotypic Data Collection

Phenotypic data of the RIL populations were collected in three seasons over two years (2021 Spring, 2021 Fall, and 2022 Spring) in the plastic greenhouse at Baima Cucumber Research Station of Nanjing Agricultural University. The experiments were conducted using a randomized complete block design (RCBD) with three replications. The phenotypic data from parental lines (IL52 and CCMC) and F_1_ individuals were only collected in 2021 Fall and 2022 Spring due to planting failure in 2021 Spring. Plant spacing was approximately 35 cm, and the row spacing was about 80 cm. 

The target traits for phenotypic data collection included ovary length (OL), ovary diameter (OD), ovary hypanthium neck width (OHN), fruit spine density (FSD), mature fruit length (MFL), mature fruit diameter (MFD), seed cavity size (SCS), mature fruit flesh thickness (MFTH), mature fruit neck length (MFNL), male flowering time (MFT), and female flowering time (FFT). Regarding ovary-related traits (OL, OD, OHN, and FSD), at least three ovaries were measured on the day of female flower blossom from each plant in each replication. FSD was recorded on a 1–4 rating scale, with 1 and 4 representing the ovary having similar dense spines as parental line IL52 and CCMC, respectively, and 2 and 3 are intermediate. The traits of OL, OD, and OHN were measured using a vernier caliper. In considered mature fruit-related traits, only the fully well-developed fruit (>35 days post pollination) were harvested for measurement from each plant. The MFT and FFT were days from sowing to the first flower anthesis.

### 2.3. Statistical Analysis of Phenotypic Data

Statistical analysis for all phenotypic data was performed in the *R/lme4* and *R/lmerTest* packages [17] with the following mixed model: *Y_ijk_* = *μ* + *G_i_* + *E_j_* + *r_jr_* + *GE_ij_* + *ε_ijr_*, where *Y* is the observed value for a given trait, *μ* for grand mean, *G* for genotype, *E* for experiments, *r* for block effects, *GE* for interaction effects of genotype and experiment, and ε for random error. The best linear unbiased predictors (BLUPs) were extracted from the model for each trait and used for QTL analysis. Broad sense heritability estimates were calculated from variance components. Spearman’s rank order correlation among traits was estimated based on the BLUP value of each RIL in the *R/corrplot* package. 

### 2.4. Genotyping and QTL Analysis

The linkage maps with 216 SSR and InDel markers for CCMC × IL52 RIL population were developed previously for QTL mapping of DM and PM resistance [14], which was used in the present study. The QTL analysis was performed in the *R/qtl* package [18]. The means of RIL in each experiment and BLUP across all experiments were used for QTL analysis. The initial whole-genome scan was performed using the “scanone” function. The detected markers with the highest LOD score from each chromosome were selected as cofactors and were applied to a multiple-QTL mapping (MQM) method using the “mqmscan” function. The LOD threshold to declare significant QTL was determined with 1000 permutation tests at *FDR < 0.05* level. For each detected QTL, the support intervals were calculated using a 1.5-LOD drop interval from the peak markers using the “lodint” function with option “expandtomarkers = T”. Nomination of QTL for various traits followed the nomenclature recommendations by Pan et al. [1] and Wang et al. [19].

### 2.5. Identification of Non-Synonymous Single-Nucleotide Polymorphisms

The clean reads of paired-end sequencing of CCMC and IL52 were aligned to cucumber reference genome 9930v3.0 from CuGenDBv2 [20] using the default parameters of Burrows–Wheeler Aligner (BWA)-Maximal Exact Match (MEM) [21]. The functional effect of single-nucleotide polymorphisms (SNPs) and small InDels was obtained and annotated using the GATK (v4.1.9) [22] pipeline and SnpEfftool (v4.3) [23].

## 3. Results

### 3.1. Phenotypic Variation of Ovary- and Fruit-Related Traits in CCMC × IL52 RIL Population

**Figure 1:**
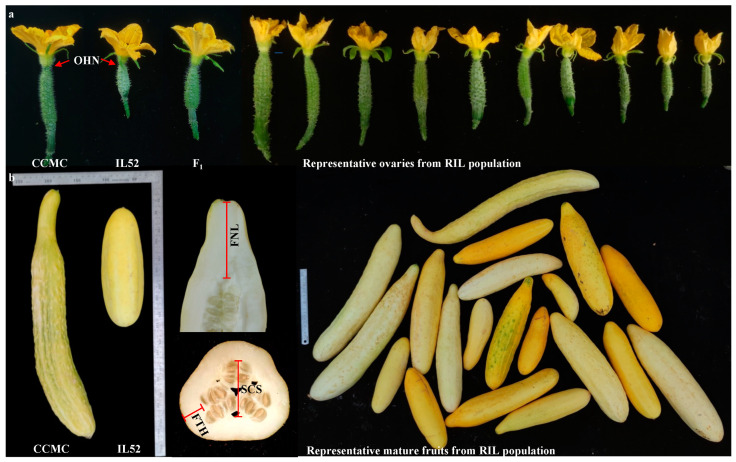
The typical images of CCMC, IL52, F_1_, and their derivates. (**a**) The ovaries, showing the differences in length, diameter, hypanthium neck width, and spine density. Red arrows mark trait of ovary hypanthium neck (OHN). (**b**) Mature fruits, showing the differences between two parental lines and the measurement criteria of fruit neck length (FNL), fruit flesh thickness (FTH), and seed cavity size (SCS).

**Figure 2:**
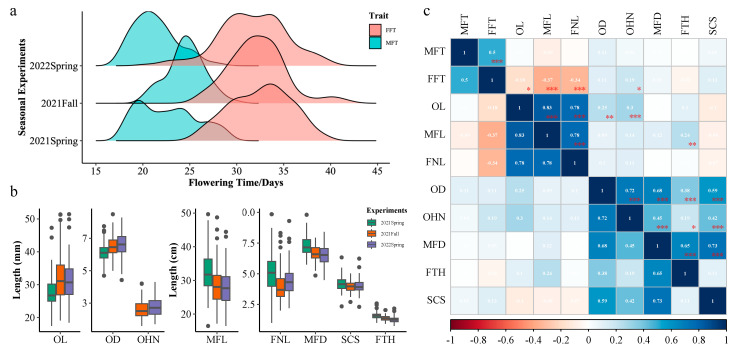
Phenotypic variation of flowering time and fruit-related traits in CCMC × IL52 RIL population across three seasonal experiments. (**a**) Frequency distribution of female flowering time (FFT) and male flowering time (MFT). (**b**) Boxplots of ovary- and fruit-related traits. The lower, middle, and upper bounds of the box in a boxplot indicate the first, second, and third quantile, respectively. The dots indicated the outliers. (**c**) Spearman’s correlation plot among 10 traits. Square colors illustrate the correlation between pairs of traits. OL, ovary length; OD, ovary diameter; OHN, ovary hypanthium neck width; MFL, mature fruit length; MFD, mature fruit diameter; FTH, fruit flesh thickness; SCS, seed cavity size; FNL, fruit neck length. * *p* < 0.05, ** *p* < 0.01, and *** *p* < 0.001.

The differences in ovaries and mature fruits between parental lines IL52 and CCMC are clearly shown in Figure 1. The phenotypic means, standard deviation, range, and estimated heritability of measured traits across three experiments are presented in Table 1. The frequency distribution depicting the genetic variations of these traits is illustrated in the ridge plot and boxplot (Figure 2a,b). The MFT and FFT of the RIL population are largely normally distributed, suggesting their quantitative nature (Figure 2a). Generally, the female flowering time of the population is later than the male flowering time (Figure 2a). All other traits, except for FSD, showed continuous distribution, also indicating their quantitative inheritance (Figure 2b). We conducted analysis of variance (ANOVA) on 10 traits and estimated their broad-sense heritability (*H^2^*). The variance components for each trait are presented in Supplementary Table S1. Genetic effects were significant for all traits. Significant effects of the seasonal experiment were found for OL, MFL, MFD, FTH, FNL, and MFT traits, while significant effects of G × E interactions were found for the traits of OL, FNL, MFT, FFT, and OHN, suggesting the performance of these traits was affected by the seasonal environments which might be due to the growing temperature differences in the spring and fall seasons (Table S1). Generally, the broad sense heritability (*H^2^*) estimates for the majority of the measured traits range from 0.66 to 0.91. However, the heritability for MFT is only 0.36, suggesting a weak heritability and that environmental factors contribute a significant proportion to the variance in MFT (Table S1). As such, QTL analysis of all traits was performed based on BLUP value across three seasonal experiments (see below).

We further analyzed the correlations between fruit traits and flowering time across experiments (Figure 2c). The Spearman’s rank order correlation showed that MFT was moderately correlated with FFT (*r_s_* = 0.5, *p* < 0.01). Among other ovary- and fruit-related traits, OL, MFL, and FNL are highly correlated (*r_s_* ranged from 0.78 to 0.83), while MFD is highly correlated with OD, FTH, and SCS (*r_s_* ranged from 0.65 to 0.73). In particular, OHN shows a high correlation with OD (*r_s_* = 0.72). These results together suggested that the genetic mechanisms might differ underlying the ovary/fruit development in longitudinal growth (length) and radial growth (diameter).

### 3.2. QTL Analysis 

**Figure 3:**
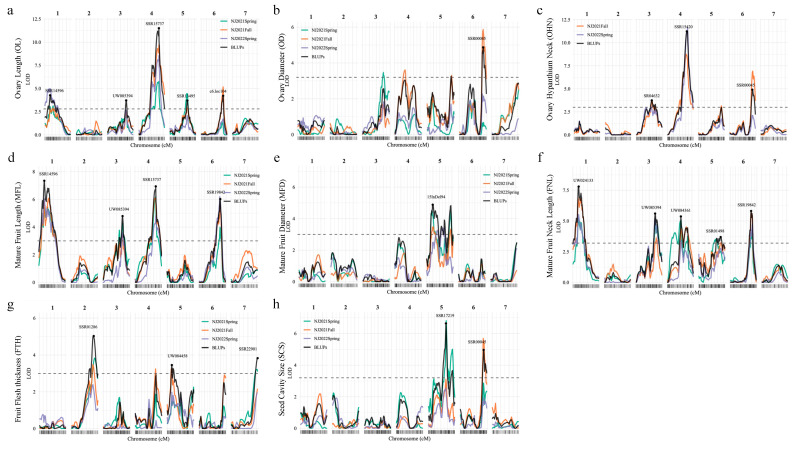
LOD profiles of ovary- and fruit-related QTL detected in CCMC × IL52 RIL population. (**a**) ovary length (OL). (**b**) ovary diameter (OD). (**c**) ovary hypanthium neck width (OHN). (**d**) mature fruit length (MFL). (**e**) mature fruit diameter (MFD). (**f**) fruit neck length (FNL). (**g**) fruit flesh thickness (FTH). (**h**) seed cavity size (SCS). The dashed horizontal line is the LOD threshold for each QTL mapping using BLUP as phenotypic value. The peak marker names are labeled on the graphs.

The linkage map for QTL mapping was constructed using 216 SSR and InDel markers which span a total map length of 653.5 cm covering 7 chromosomes [14]. A whole-genome scan for QTL was first conducted using the MQM method in *R/qtl*. We performed the QTL analysis using the means from each experiment and BLUPs extracted from three experiments and found that for most of the traits, there were no significant differences in QTL detection utilizing means and BLUPs. Thus, we only reported the QTL detected by BLUP with its peak position, 1.5-LOD support intervals, explained variances, and additive effects herein, which are illustrated in Figure 3, Figure S2, and Table 2. In total, 27 QTL for 11 traits were detected, which were distributed on 7 chromosomes. These QTL explained 3.61–43.98% of the phenotypic variance (PV). The detailed information is described below.

#### 3.2.1. QTL Mapping for Ovary-Related Traits

For ovary-related traits (OL, OD, OHN, and FSD), a total of 10 QTL were identified on chromosomes (Chr) 1, 3, 4, 5, and 6, explaining 4.8–44.0% phenotypic variation (Figure 3a–c; Table 2). Four QTL for OL were identified on Chr 1, 3, 4, and 5, explaining a total of 62.76% PV. Among them, the major-effect QTL is *qOL4.1,* explaining 25.94% PV. All QTL had a negative additive effect, indicating alleles from IL52 contributed to the decrease in fruit elongation (Table 2). Only one QTL for OD and FSD were identified on Chr 6 (*qOD6.1* and *qFSD6.1*), and both were located in the similar interval of 25.37–30.21 Mbp, explaining 13.19% and 43.98% PV, respectively. Three QTL for OHN were identified on Chr 3, 4, and 6, with the major-effect QTL (*qOHN4.1*, PV = 29.85%) co-localized with *qOL4.1* and the minor-effect QTL (*qOHN6.1*, PV = 16.05) co-localized with *qOD6.1*. This might suggest that the OHN width is the net outcome of the ovary elongation and radial growth. 

Particularly, we noticed that only one significant QTL, *qFSD6.1,* was detected for FSD with a rigorous LOD (LOD = 18.87) flanking by SSR01234 and SSR18251 (~2.0 Mb) (Table 2). This may hint that FSD is regulated by a single gene. Therefore, we manually designate the ovary with an FSD rating scale of 1 or 2 as sparse-spined and those with a rating scale of 3 or 4 as dense-spined. As a result, in the RIL population, a total of 67 lines and 82 lines exhibited sparse spines and dense spines, respectively, on the ovary surface, which fit the 1:1 Mendel’s segregation ratio (*p* = 0.216 in the chi-square test). This result supports that FSD is regulated by a single inherited gene. Thus, we reconstructed the genetic linkage map for Chr 6 by treating FSD as a phenotypic marker. We then mapped the *qFSD6.1* between marker SSR01234 and CSWCT5B with the physical interval of 566 kb from 28.231 to 28.797 Mb on Chr 6 according to 9930 v3.0 genome assembly (Figure S2A). This result is further confirmed by examining the phenotypic data of recombinants defined by markers SSR01234, CSWCT5B, and SSR18251. Notably, the 566 kb candidate region covered the previously identified QTL *fsd6.2* and *Csgl3*, both of which have been proposed to regulate high fruit spine density in natural cucumber populations [24,25,26,27].

#### 3.2.2. QTL Mapping for Fruit-Related Traits

For mature fruit-related traits, a total of 15 QTL were detected located in all seven cucumber chromosomes, which could explain observed PV ranging from 3.61 to 18.59% (Figure 3d–h; Table 2). Notably, according to the 1.5 LOD support interval, co-localizations were observed among those QTL detected for MFL, FNL, and OL: *qMFL1.1/qFNL1.1/qOL1.1*, *qMFL3.1/qFNL3.1/qOL3.1*, *qMFL4.1/qFNL4.1/qOL4.1*, *qFNL5.1/qOL5.1*, and *qMFL6.1/qFNL6.1/qOL6.1*. This may indicate that the elongation growth of the fruit neck length is generally under the regulation of fruit elongation. Moreover, these QTL contributed a negative additive effect, suggesting that the CCMC allele is responsible for elongation growth. 

Furthermore, we investigated the mature fruit diameter (MFD) by dissecting it into the fruit flesh thickness (FTH) and seed cavity size (SCS). One, three, and two QTL for MFD, FTH, and SCS, respectively, were identified, explaining 6.89−18.59% PV. Based on their chromosomal locations, the QTL detected on Chr 5 for MFD, FTH, and SCS were co-localized (*qMFD5.1/qFTH5.1/qSCS5.1*) and contributed a positive additive effect, indicating alleles from IL52 contributed to increased fruit radial growth. Additional QTL identified for FTH were *qFTH2.1* (PV = 10.98%) and *qFTH7.1* (PV = 8.20%), and that for SCS was *qSCS6.1* (PV = 13.55%). In particular, *qSCS6.1* had a negative additive effect, suggesting it may neutralize the genetic effect of fruit radial growth contributed by *qFTH2.1* and *qFTH7.1*. Moreover, the QTL *qSCS6.1* was co-localized with ovary diameter QTL *qOD6.1* and ovary hypanthium neck width QTL *qOHN6.1*, suggesting the non-synchronized radial growth between the endocarp and mesocarp of cucumber fruit. 

#### 3.2.3. QTL Mapping for Flowering Time

In the RIL population, two major-effect QTL were detected for MFT (*qmft1.1*) and FFT (*qfft1.1*), which explained 11.47% and 37.25% PV, respectively. Both QTL *qmft1.1* and *qfft1.1* are located in Chr 1 but at different genomic positions spanning 1.5-LOD support intervals at Chr 1: 21.06–25.34 Mb and Chr 1: 2.95–4.23 Mb, respectively (Table 2; Figure S2B). The IL52 allele contributed to a delay in both male and female flowering time, displaying a positive additive effect. 

To investigate the relationship between flowering time and the position of the first flower node, we measured the male/female first flower node (MFFN and FFFN) for the two parental lines and RIL population. However, we did not detect any significant differences; the MFFN are 2–4 nodes and FFN are 7–10 nodes for both IL52 and CCMC (data not shown). This suggests that the delayed flowering time of male and female flowering time in IL52 is not likely associated with the position of the flower node on the stem. 

### 3.3. Candidate Region of qOHN4.1 in the RIL Population

**Figure 4:**
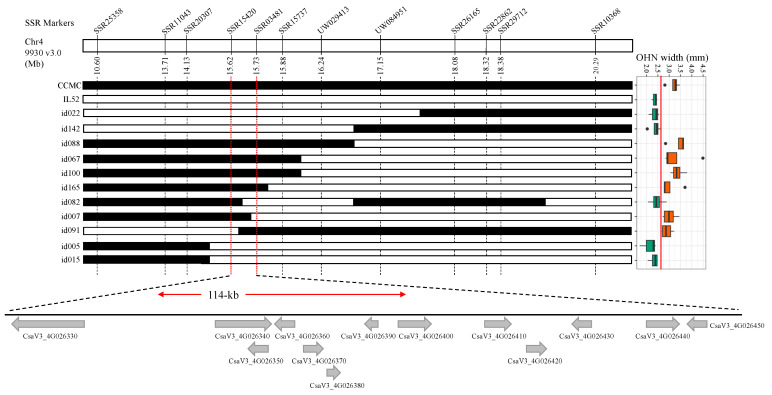
Refinement of map location of major QTL *qOHN4.1* for ovary hypanthium neck in cucumber. The number beneath the markers indicates their physical position. The black boxes represent the homozygous genotypes of CCMC. The white boxes represent the homozygous genotypes of IL52. The genotypes of 11 representative RIL lines are shown. The boxplots at right shows their phenotypic data collected from all seasonal experiments. The predicted genes in 9930 v3.0 draft genome from candidate 114-kb region are shown as grey arrows.

For the trait of OHN, the QTL *qOHN4.1* is the major-effect QTL and is co-localized with *qOL4.1*, *qMFL4.1*, and *qFNL4.1*, which are located in a region of ~3.02 Mb (1.5−LOD interval) delimited by SSR20307 and UW084951 (Figure 3; Table 2). Among 155 RILs, a total of 25 recombinants were identified using these two flanking markers. We further examined the genotypes of 25 recombinants with 4 additional SSR markers in this region (SSR15420, SSR03481, SSR15737, and UW029413). Among them, 9 RILs have a genetic background, with the small-effect QTL *qOHN3.1* and *qOHN6.1* candidate regions harboring homozygous alleles as parental line CCMC. The OHN data of the 9 RILs from all seasonal experiments were gathered to represent its distribution via boxplot (Figure 4). Based on the genotypes and phenotypes of 9 RILs, the *qOHN4.1* was narrowed down to a region defined by markers SSR15420 and SSR03481 that were ~114 kb apart (Figure 4). According to the 9930 v3.0 draft genome, 13 genes were predicted in this region (Table 3); among them, two with transcription factor (TF) genes (*CsaV3_4G026430* and *CsaV3_4G026450*) and three genes (*CsaV3_4G026370/380* and *CsaV3_4G026440*) have been reported to be associated with cell apoptosis, proliferation, and differentiation. 

The two parental lines, IL52 and CCMC, were previously re-sequenced using Illumina HiSeq 2000 [14]. We assessed the polymorphisms between the two parental lines and identified 6 out of 13 genes harboring non-synonymous SNPs. These genes include *CsaV3_4G026360,* encoding late embryogenesis abundant protein; *CsaV3_4G026370,* encoding ERBB-3 binding protein 1; *CsaV3_4G026400/410/420,* encoding a cluster of Pyruvate decarboxylase; and *CsaV3_4G026450,* encoding MYB-like TF (Table 3). Based on the detected polymorphisms and their predicted gene function, we propose that *CsaV3_4G026370* and *CsaV3_4G026450* might be the best candidate underlying the co-localized QTL *qOHN4.1/qOL4.1/qMFL4.1/qFNL4.1*.

## 4. Discussion

### 4.1. The Potential Pleiotropic Effect of qOHN4.1 for Ovary Development

In the present study, using the CCMC × IL52 RIL population, we identified 27 QTL for 11 traits, including flowering time and ovary- and fruit-related traits (Table 2). The trait of ovary hypanthium neck (OHN) width was noticed during hand pollination in the 2021 Spring season: narrow OHNs were easily broken. This trait has not been reported in other populations. We then collected the phenotypic data in the other two seasonal experiments 2021 Fall and 2022 Spring, which presented in continuous distributions and showed a high correlation with ovary diameter (*r_s_* = 0.72). QTL mapping revealed a major QTL, *qOHN4.1,* was repeatably detected in two experiments which explained >29% phenotypic variance (Figure 3). We further narrowed down the candidate region of *qOHN4.1* in a 114-kb interval, which is delimited by SSR markers SSR15420 and SSR03481. According to the 9930 v3.0 draft genome assembly, a total of 13 candidate genes were annotated within this region. Among them, six genes exhibited non-synonymous single-nucleotide polymorphisms (nsSNPs) between CCMC and IL52. Notably, two genes, *CsaV3_4G026370* encoding ERBB-3 binding protein and *CsaV3_4G026450* encoding myb-like transcription factors, emerged as the most possible candidates underlying *qOHN4.1* (Figure 3). Additional work is needed to confirm the candidate genes, such as developing near-isogenic lines (NILs) to identify more recombinants for further narrowing down the candidate region.

The QTL *qOHN4.1* was found co-localized with the QTL identified for ovary- and fruit-related traits, including *qOL4.1*, *qMFL4.1*, and *qFNL4.1* (Table 3). This QTL was assigned as a consensus *FS4.1* [1] due to the fact that it has been reported in a number of populations, including Gy14 × 9930 RILs [2], S1000 × S1002 F_2:3_ families [28], WI7167 × WI7200 F_2:3_ families [29], and CS-PMR1 × Santou RILs [30]. The cucumber *FS4.1* is syntenic to the melon *CmFSI8,* whose underlying causal gene is *CmOFP1a/CmOFP13* [1,7,31]. Thus, the *CsOFP1a* might be considered a top candidate for *FS4.1*. Given the fact that *CsOFP1a* (*CsaV3_4G027080*) is approximately 580-kb distance from the candidate region of *qOHN4.1*, we may hypothesize that either the *FS4.1* is rich in fruit length-regulated genes or *qOHN4.1* has a potential pleiotropic effect that mediates OL and MFL as well.

In addition, our previous study using the same population (CCMC × IL52 RIL) revealed the genetic inheritance of cucumber fruit apex at commercial harvest stages (12–15 days post anthesis ) [16]. A total of four QTL were identified for fruit apex index (*fai*, defined by fruit apex length/fruit apex diameter), including *Ofai3.1*, *Ofai3.2*, *Ofai3.3*, and *Ofai4.1*. Among them, the 1.5-LOD interval physical position of *Ofai4.1* is co-localized with *qOHN4.1*, suggesting that this locus involved in shaping the cucumber fruit apex may be relevant to regulating the OHN width [16]. In particular, by comparing the phenotypic data of fruit apex index, a wider OHN normally has a longer apex. Since the apex length is the component of ovary length, it provided a reasonable explanation for the co-localization of *qOHN4.1* with *Ofai4.1*, *qOL4.1*, and *qMFL4.1.* The cucumber fruit apex structure is very similar to the pointed-tip structure of tomato fruit. The tomato flower is hypogynous, and the ovary is not coated by the hypanthium. The pointed tip of an ovary at the distal end would promote its development into a heart-shaped tomato fruit. The candidate gene regulating the presence and absence of a pointed tip has been identified through a GWAS study that is *POINTED TIP* (*PT*) gene encoding a C_2_H_2_-type zinc finger transcription factor [32]. The *PT* gene functions to suppress the formation of a pointed tip by downregulating the transcription of the *FRUITFULL2* (*FUL2*) gene that participates in the auxin transportation pathway [32]. Further analysis is necessary to determine whether *qOHN4.1* promoted the growth of OHN and cucumber fruit apex in coordination with other fruit elongation genes or hormone-related genes.

### 4.2. QTL for Fruit Size Variation in IL52 and CCMC

In this study, we phenotyped ovary-related traits (OL, OD, OHN, and FSD) and mature fruit-related traits (MFL, MFD, FNL, FTH, and SCS). Except for mature fruit diameter, inheritance of all ovary- and fruit-related traits exhibited continuous variation, and their polygenic nature with multiple QTL was identified (Table 2). The interaction effect between genotypes and seasonal environments was found to be significant for ovary length and fruit neck length (Table S1). FL and FD were found to have moderate to high correlation in many other populations, including Gy14 × 9930 RIL, WI2757 × TL RIL, WI7088 × Coolgreen RIL, and WI7120 × 9930 F_2:3_ families [1,2,33]; this suggests the common QTL/genes in regulated cell division and expansion in longitude and radial direction in these lines. In contrast, trait correlations in CCMC × IL52 RIL clearly showed the potential independence of genetic mechanisms underlying elongation and radial growth. Length-related traits, such as OL, MFL, and FNL, are highly correlated with *r_s_* ranging from 0.78−0.83, while diameter-related traits are grouped together (Figure 2). No significant correlations were identified between length-related traits and diameter-related traits (Figure 2). 

In addition, we found that SCS and FTH, each a component of MFD, are highly correlated with MFD with *r_s_* = 0.73 and *r_s_* = 0.65, respectively. However, no significant correlation was detected between SCS and FTH (Figure 2). This could be reflected by the QTL analysis results that only one co-localized QTL were detected for SCS and FTH, that is *qSCS5.1* and *qFTH5.1*. This is similar to that found in WI7088 × Coolgreen RIL and WI7120 × 9930 F_2:3_ families, while WI7088 and WI7120 both have large, hollow seed cavities [33]. In addition, SCS and FTH in Gy14 × 9930 RIL population display a negative correlation (*r_s_* = −0.75) [2]. All these findings suggested a non-synchronized radial growth of the endocarp and mesocarp of a cucumber fruit of IL52.

### 4.3. Flowering Time QTL in IL52 and CCMC

The male and female flowering times of parental lines (CCMC and IL52) and RIL population plants are affected by the environment. Plants planted in the spring season displayed a delayed flowering time compared with the fall season (Figure 2). This might be due to the low accumulated temperature in the early spring season compared with late summer. The female flower of IL52 blossomed approximately 9−11 days later than that of CCMC (Table 1). Only one major-effect QTL for MFT and FFT was detected (*qMFT1.1* and *qFFT1.1*). So far, six consensus flowering time QTL have been identified, including *FT1.1*, *FT5.1*, *FT6.1*, *FT6.2*, *FT6.3*, and *FT6.4* [33]. Except for *FT1.1,* which has been detected in multiple mapping populations, the other consensus *FT* QTL were only detected in very few populations, including the cross with wild Hardwickii cucumber, semi-wild Xishuangbanna cucumber, and Sikkim cucumber [5,29,33,34,35]. 

Two large deletions upstream from the *FT1.1* locus were believed to be associated with the regulation of the *FLOWERING LOCUS T* (*FT*) gene, which is responsible for the earlier flowering adaption of cucumber [36,37]. In the present study, the peak marker for *qMFT1.1* is the end marker in the linkage map of Chr 1; the linkage map did not cover the 26–32 Mb region where *FT* resided (Figure S2B). Thus, we are not able to rule out the possibility that the *FT* gene also contributes to the male flowering time *qMFT1.1* as well. In contrast, the female flowering time *qFFT1.1* is located at the beginning of Chr 1, suggesting a novel *ft* locus that exists in the CCMC × IL52 RIL population. Further genetic fine mapping is required to reveal its candidate gene and its relationship with the *FT* gene.

## Figures and Tables

**Table 1:** Phenotypic means and standard deviation (SD) of horticulturally important traits in CCMC, IL52, their F_1_, and RIL populations in three experiments.

Phenotypes		CCMC (2021 Fall)	CCMC (2022 Spring)	IL52 (2021 Fall)	IL52 (2022 Spring)	F_1_ (2021 Fall)	F_1_ (2022 Spring)	RIL Population **	
Traits	Abbr. *	Mean ± SD	Mean ± SD	Mean ± SD	Mean ± SD	Mean ± SD	Mean ± SD	Mean ± SD	Range
Flower time	MFT	26.50 ± 1.73	19.33 ± 3.06	27.25 ± 3.40	22.67 ± 0.58	NA	20.00 ± 0.00	22.94 ± 3.52	14.00–35.00
	FFT	29.25 ± 2.87	25.00 ± 0.00	38.00 ± 4.90	36.33 ± 5.69	31.33 ± 2.16	26.67 ± 1.53	32.17 ± 4.49	18.00–46.00
Ovary trait	OL	40.34 ± 5.68	44.84 ± 9.53	19.03 ± 1.48	19.95 ± 1.07	33.32 ± 2.60	NA	31.68 ± 7.69	12.43–67.59
	OD	6.36 ± 0.26	6.67 ± 0.21	6.35 ± 0.42	6.13 ± 0.38	6.31 ± 0.63	NA	6.58 ± 0.82	2.04–9.83
	OHN	3.27 ± 0.16	3.14 ± 0.47	2.39 ± 0.14	2.33 ± 0.15	2.57 ± 0.39	NA	2.72 ± 0.69	1.24–8.07
	FSD	4	4	1	1	2	2	2.19 ± 0.88	1.00–3.00
Mature fruit trait	MFL	43.73 ± 3.13	45.88 ± 8.03	22.25 ± 0.35	19.25 ± 0.35	32.37 ± 5.58	24.23 ± 3.16	28.07 ± 6.02	12.20–56.00
	MFD	7.49 ± 0.06	6.52 ± 0.80	7.83 ± 0.74	6.74 ± 0.79	6.97 ± 0.64	7.12 ± 0.86	6.58 ± 0.89	4.10–9.60
	FTH	1.38 ± 0.10	1.22 ± 0.29	1.68 ± 0.25	1.39 ± 0.30	1.38 ± 0.24	1.53 ± 0.25	1.31 ± 0.28	0.50–2.57
	SCS	4.58 ± 0.15	4.02 ± 0.64	4.15 ± 0.21	4.29 ± 0.30	4.12 ± 0.30	4.02 ± 0.50	3.95 ± 0.60	2.30–6.89
	FNL	9.07 ± 1.31	8.09 ± 0.99	2.65 ± 0.21	2.47 ± 0.75	5.98 ± 0.90	5.61 ± 4.67	4.17 ± 1.42	1.00–11.00

* MFT, Male flowering time, days; FFT, Female flowering time, days; OL, Ovary length, mm; OD, Ovary diameter, mm; OHN, Ovary hypanthium neck width, mm; FSD, Fruit spine density; MFL, Mature fruit length, cm; MFD, Mature fruit diameter, cm; FTH, Fruit flesh thickness, cm; SCS, Seed cavity size, cm; FNL, Fruit neck length, cm. ** The mean and standard deviation (SD) is calculated based on the phenotypic data collected from three seasonal experiments. NA: Not Applied/Not Available.

**Table 2:** Summary of the quantitative trait loci (QTL) detected in the RIL mapping population derived from the cross of IL52 and CCMC based on the BLUP value.

Trait	QTL	Chr	LOD	Peak Marker	Position (in cm)	1.5–Lod Interval (9930 v3.0)				Additive Effect	PV/100%	Total PV/100%
						Left Marker	Physical Position (Mb)	Right Marker	Physical Position (Mb)			
OL	*qOL1.1*	1	4.27	SSR14596	18.5	UW044821	1.72	UW083977	11.71	−1.196	7.422	62.76
	*qOL3.1*	3	3.72	UW085394	84.6	SSR20270	28.14	SSR18311	31.85	−0.977	4.806	
	*qOL4.1*	4	11.51	SSR15737	66.3	SSR20307	14.13	UW029413	16.24	−2.228	25.94	
	*qOL5.1*	5	3.70	SSR13495	70.9	SSR15321	21.13	UW013295	25.13	−0.980	4.813	
	*qOL6.1*	6	4.21	c6.loc104	104.0	SSR16005	23.33	SSR00045	27.53	−1.432	10.49	
OD	*qOD6.1*	6	4.87	c6.loc108	108.0	SSR19970	25.37	SSR18251	30.21	−0.141	13.19	28.19
OHN	*qOHN3.1*	3	3.75	c3.loc70	70.0	SSR03409	9.51	SSR07131	34.16	0.129	7.214	53.11
	*qOHN4.1*	4	11.23	c4.loc64	64.0	SSR20307	14.13	UW084951	17.14	−0.264	29.85	
	*qOHN6.1*	6	4.91	c6.loc108	108.0	SSR19970	25.37	SSR18251	30.21	−0.188	16.05	
FSD	*qFSD6.1*	6	18.87	CSWCT5B	112.6	SSR01234	28.23	SSR18251	30.21	−0.447	43.98	43.98
MFL	*qMFL1.1*	1	7.34	SSR14596	18.5	SSR04304	2.19	UW062539	8.91	−1.641	12.63	54.28
	*qMFL3.1*	3	4.79	c3.loc86	86.0	SSR10697	29.66	SSR07131	34.16	−1.189	6.594	
	*qMFL4.1*	4	6.93	SSR15737	66.3	SSR20307	14.13	UW084951	17.14	−1.715	14.04	
	*qMFL6.1*	6	6.02	c6.loc98	98.0	C31	21.60	SSR15316	26.60	−1.445	9.251	
FNL	*qFNL1.1*	1	7.80	UW024133	19.9	SSR05793	3.14	SSR10134	8.22	−0.401	14.3	58.31
	*qFNL3.1*	3	5.61	c3.loc86	86.0	SSR23725	29.95	SSR07131	34.16	−0.257	5.82	
	*qFNL4.1*	4	5.37	UW084361	86.0	SSR16892	6.38	UW084951	17.14	−0.385	13.27	
	*qFNL5.1*	5	3.69	c5.loc88	88.0	SSR18792	15.22	UW084826	31.16	−0.206	3.619	
	*qFNL6.1*	6	5.81	c6.loc98	98.0	SSR17818	23.01	SSR20599	27.41	−0.292	7.008	
MFD	*qMFD5.1*	5	4.89	c5.loc24	24.0	15InDel55	2.92	UW085322	29.62	0.159	13.42	13.42
FTH	*qFTH2.1*	2	5.02	c2.loc60	60.0	UW085249	14.54	SSR22653	19.07	−0.042	10.98	30.44
	*qFTH5.1*	5	3.45	UW084458	18.4	15InDel40	2.13	UW084826	31.16	0.033	6.894	
	*qFTH7.1*	7	3.83	SSR22901	63.2	SSR13188	18.50	SSR22901	22.21	0.038	8.204	
SCS	*qSCS5.1*	5	6.63	SSR17219	69.9	UW084138	22.36	SSR16068	24.70	0.126	18.59	32.25
	*qSCS6.1*	6	4.97	c6.loc108	108.0	SSR19970	25.37	SSR23639	30.94	−0.107	13.55	
MFT	*qMFT1.1*	1	4.08	c1.loc88	88.0	UW085274	21.06	SSR16841	25.34	0.241	11.47	11.46
FFT	*qFFT1.1*	1	15.39	c1.loc16	16.0	SSR20760	2.95	UW024133	4.23	1.338	37.25	37.25

**Table 3:** The annotation of predicted 13 genes in the candidate region of ovary hypanthium neck (*qOHN4.1*) locus according to 9930 v3.0 draft genome.

Gene ID	nsSNPs *	Gene Annotation	Predicted Function
CsaV3_4G026330	0	AMP deaminase	Promote cell activity
CsaV3_4G026340	0	Chlorophyll synthase	Photosystem
CsaV3_4G026350	0	Late embryogenesis abundant protein B19.1A	Abiotic stress response
CsaV3_4G026360	1	Late embryogenesis abundant protein B19.3	Abiotic stress response
CsaV3_4G026370	1	ERBB-3 BINDING PROTEIN 1	Apoptosis, cell proliferation, and differentiation
CsaV3_4G026380	0	ERBB-3 BINDING PROTEIN 1	Apoptosis, cell proliferation, and differentiation
CsaV3_4G026390	0	Photosystem II D2 protein	Photosystem
CsaV3_4G026400	4	Pyruvate decarboxylase	Abiotic and biotic stress response
CsaV3_4G026410	1	Pyruvate decarboxylase	Abiotic and biotic stress response
CsaV3_4G026420	4	Pyruvate decarboxylase	Abiotic and biotic stress response
CsaV3_4G026430	0	BHLH domain-containing protein	Transcription factor
CsaV3_4G026440	0	BAG family molecular chaperone regulator 4	Diverse cellular processes, including apoptosis, proliferation, differentiation, and stress signaling
CsaV3_4G026450	3	HTH myb-type domain-containing protein	Transcription factor

* nsSNPs: non-synonymous single-nucleotide polymorphisms.

## Data Availability

Not applicable.

## Supplementary Materials

The following supporting information can be downloaded at: https://www.mdpi.com/article/10.3390/genes14061133/s1. Figure S1: An anatomy of epigynous flower and female cucumber flower; Figure S2: Genetic mapping for fruit spine density (FSD) and QTL mapping for male/female flowering time (MFT/FFT); Table S1: Analysis of variance (ANOVA) of CCMC × IL52 RIL population and estimation of broad-sense heritability.
