# Supplementary material for: QTL Mapping for Ovary- and Fruit-Related Traits in Cucumis sativus-C. hystrix Introgression Line IL52
Source: Genes (Basel). 2023 May 23;14(6):1133. doi: 10.3390/genes14061133 (PMC10297961; doi:10.3390/genes14061133)
Supplement: Supplementary file 1 [file genes-14-01133-s001.zip › FigureS2.pdf]

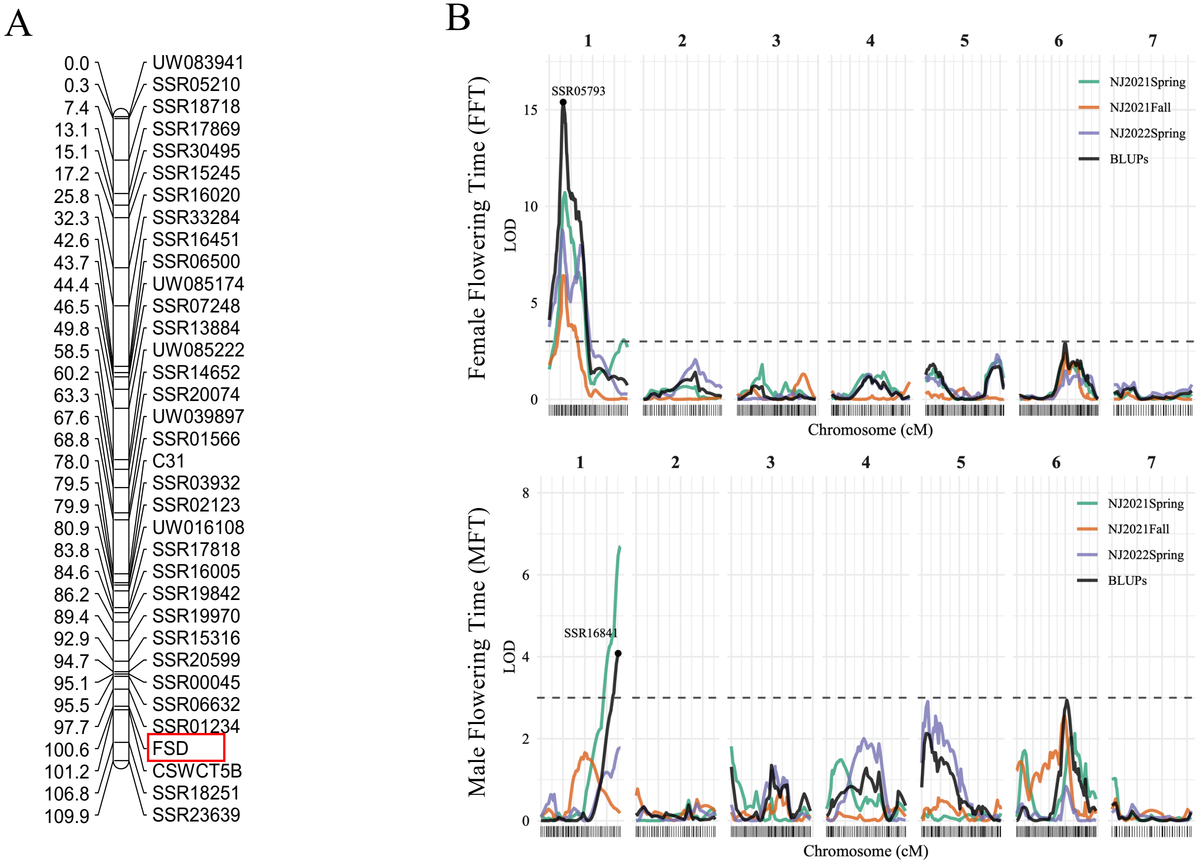

Figure S2. Genetic mapping for fruit spine density (FSD) and QTL mapping for male/female flowering time (MFT/FFT). The dashed horizontal line in (**B**) is LOD threshold for each QTL mapping using BLUP as phenotypic value. The peak marker names are labeled on the graphs.
