# Supplementary material for: QTL Mapping for Ovary- and Fruit-Related Traits in Cucumis sativus-C. hystrix Introgression Line IL52
Source: Genes (Basel). 2023 May 23;14(6):1133. doi: 10.3390/genes14061133 (PMC10297961; doi:10.3390/genes14061133)
Supplement: Supplementary file 1 [file genes-14-01133-s001.zip › FigureS1.pdf]

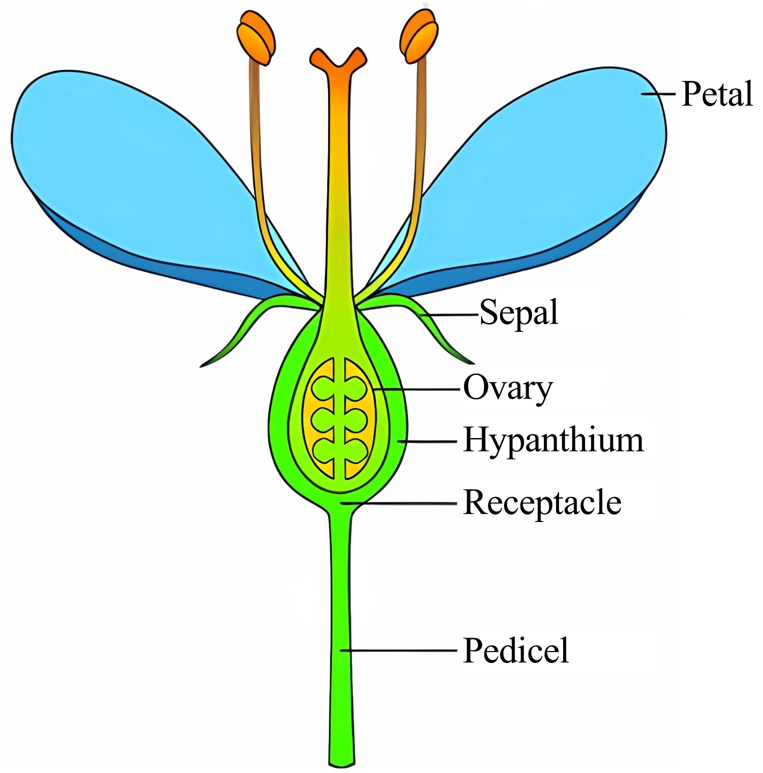

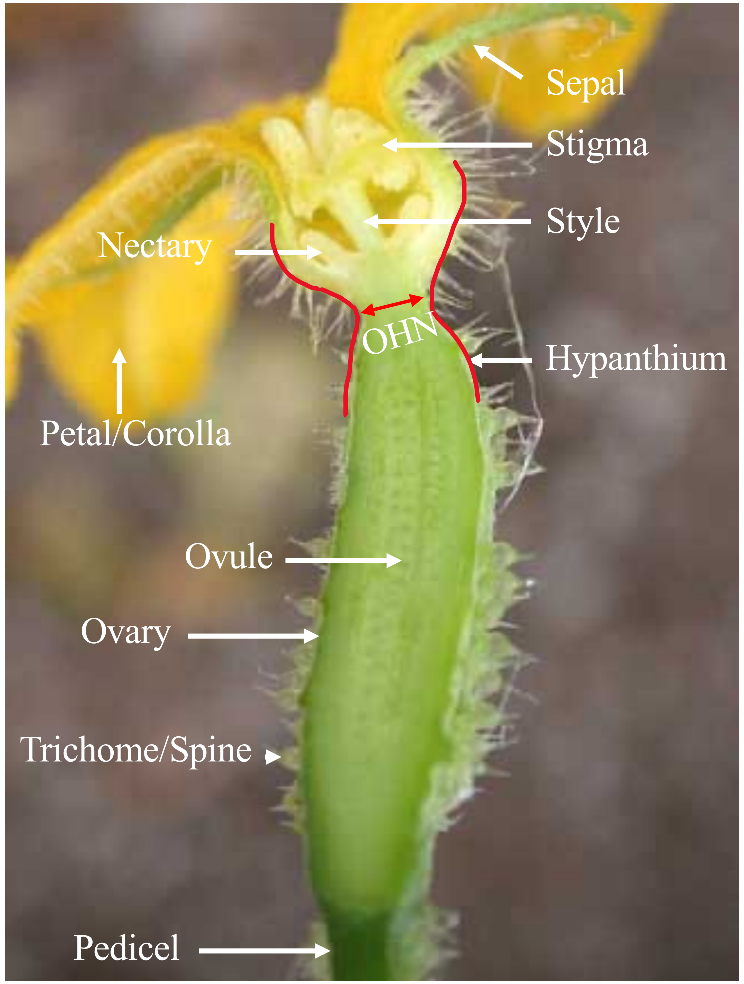

Figure S1. An anatomy of epigynous flower and cucumber female flower. The main structures are annotated on the drawings and photos. The hour-glass shaped of hypanthium is marked in red lines. OHN, ovary hypanthium neck.
